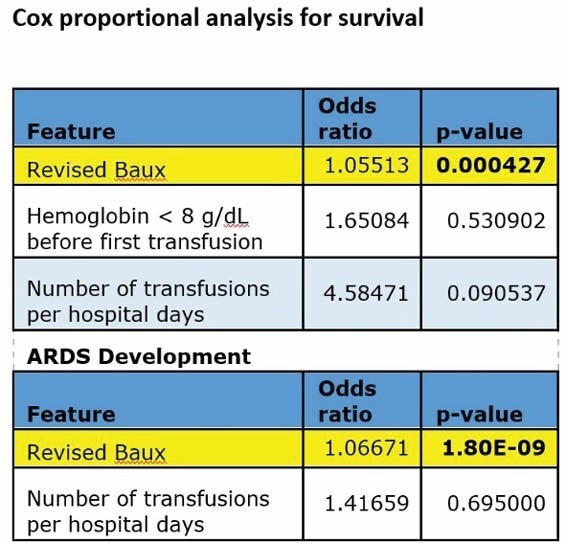# 92 Effect of Blood Transfusion on Outcomes After Major Burns

**DOI:** 10.1093/jbcr/irad045.065

**Published:** 2023-05-15

**Authors:** Maryum Merchant, Scott Hu, Malcolm Smith, stella Cohen, Geethika Janga, Saineha Maddineni

**Affiliations:** University of California, Los Angeles, Westlake Village, California; University of California, Los Angeles, Los Angeles, California; UCLA, Santa Monica, CA, California; University of California Los Angeles, Encino, California; University of California, Los Angeles, Los Angeles, California; University of California, Los Angeles, Los Angeles, California; University of California, Los Angeles, Westlake Village, California; University of California, Los Angeles, Los Angeles, California; UCLA, Santa Monica, CA, California; University of California Los Angeles, Encino, California; University of California, Los Angeles, Los Angeles, California; University of California, Los Angeles, Los Angeles, California; University of California, Los Angeles, Westlake Village, California; University of California, Los Angeles, Los Angeles, California; UCLA, Santa Monica, CA, California; University of California Los Angeles, Encino, California; University of California, Los Angeles, Los Angeles, California; University of California, Los Angeles, Los Angeles, California; University of California, Los Angeles, Westlake Village, California; University of California, Los Angeles, Los Angeles, California; UCLA, Santa Monica, CA, California; University of California Los Angeles, Encino, California; University of California, Los Angeles, Los Angeles, California; University of California, Los Angeles, Los Angeles, California; University of California, Los Angeles, Westlake Village, California; University of California, Los Angeles, Los Angeles, California; UCLA, Santa Monica, CA, California; University of California Los Angeles, Encino, California; University of California, Los Angeles, Los Angeles, California; University of California, Los Angeles, Los Angeles, California; University of California, Los Angeles, Westlake Village, California; University of California, Los Angeles, Los Angeles, California; UCLA, Santa Monica, CA, California; University of California Los Angeles, Encino, California; University of California, Los Angeles, Los Angeles, California; University of California, Los Angeles, Los Angeles, California

## Abstract

**Introduction:**

Severely burnt patients frequently receive blood transfusions to treat anemia, increase blood volume and improve wound healing. However, transfusion is not a safe prescription as it is associated with infections and immunosuppression.

**Aim:**

Understand blood transfusion practices and assess patient transfusion-related outcomes.

**Methods:**

A single-center retrospective review of 113 ICU patients with >20% TBSA burns from Jan 2014 to June 2022 was performed. Data collected: Age, type of burns, prior use of blood thinners, # of burn operations, hematocrit level pre and post-last transfusion, # of infections, AKI, ARDS during hospitalization, and hospital and ICU length of stay (LOS)

**Statistical analysis:**

For univariate analysis comparing groups below to the above median of transfusions per hospital stay, a Fisher test was used to compare proportions, and either a t-test or a Wilcoxon test was used to compare continuous features. Cox proportional hazards model and logistic regression were used to analyze the survival and ARDS in multivariate analysis.

**Results:**

Demographics

Out of 113 patients:

•94 survived admission

•54 had a concurrent inhalational injury

•21 developed ARDs

•38 developed AKI

•95 received at least 1 blood product

Median Data:

•Age: 43 years

•TBSA: 34%

•Hospital LOS: 37 days

•ICU LOS: 18 days

Mean Data:

PRBC transfusion/patient: 12

PRBC per 1% TBSA burnt: 0.36

**Univariate analysis comparing the median # of transfusions per hospital LOS.**

Patients who had more transfusions were mostly men, had higher TBSA burns, less inhalational injury, longer ICU and hospital stays, and more burn surgeries. They also had higher hemoglobin levels before their first transfusion and more infections. There was no difference in terms of mortality, AKI, or ARDS.

Having anemia (defined here as hemoglobin < 8g/dL) before burn injury did not affect survival. After controlling for the revised Baux score and hemoglobin before initial transfusion, more transfusions per hospital day were associated with decreased survival and increased infections.

There was no association between ARDS in multivariate analysis and increased transfusions.

**Conclusions:**

The number of transfusions per hospital day was associated with decreased survival, although not meeting statistical significance (likely due to the small sample size). Hemoglobin < 8 g/dL before burns was not associated with decreased survival in multivariate analysis. On exploratory analysis, more transfusions per hospital day were significantly associated with increased infection, offering a possible reason for the negative survival association.

**Applicability of Research to Practice:**

Understanding blood transfusion practices and outcomes improve the utilization of blood in severely burnt patients.